# Maternal and neonatal outcomes in mothers with diabetes mellitus in qatari population

**DOI:** 10.1186/s12884-021-04124-6

**Published:** 2021-09-24

**Authors:** Mohammad A. A. Bayoumi, Razan M. Masri, Nada Y. S. Matani, Mohamed A. Hendaus, Manal M. Masri, Prem Chandra, Lisa J. Langtree, Sunitha D’Souza, Noimot O. Olayiwola, Saad Shahbal, Einas E. Elmalik, Mohamed S. Bakry, Ashraf I. Gad, Ravi Agarwal

**Affiliations:** 1 Neonatal Intensive Care Unit (NICU), Women’s Wellness and Research Center (WWRC), Hamad Medical Corporation (HMC), Doha, P.O. Box 3050, Qatar; 2 Department of Medical Education, Hamad Medical Corporation (HMC), Doha, Qatar; 3 Pediatric Department, Sidra Medicine, Doha, Qatar; 4 Al-Ahli Hospital, Doha, Qatar; 5 Medical Research Center, Hamad Medical Corporation (HMC), Doha, Qatar; 6 Medical Records Department, Women’s Wellness and Research Center (WWRC), Hamad Medical Corporation (HMC), Doha, Qatar; 7 Corporate Communications Department, Hamad Medical Corporation (HMC), Doha, Qatar; 8 Obstetrics and Gynecology Department, Faculty of Medicine, Fayoum University, Fayoum, Egypt

**Keywords:** Gestational Diabetes Mellitus, Women, Newborn, Infant of Diabetic Mother, Qatari

## Abstract

**Abstract:**

**Background:**

Diabetes Mellitus (DM) is a major cause of maternal, fetal, and neonatal morbidities. Our objective was to estimate the effect of both pre-pregnancy and gestational DM on the growth parameters of newborns in the Qatari population.

**Methods:**

In this population-based cohort study, we compared the data of neonates born to Qatari women with both pre-pregnancy and gestational diabetes mellitus in 2017 with neonates of healthy non-diabetic Qatari women.

**Results:**

Out of a total of 17020 live births in 2017, 5195 newborns were born to Qatari women. Of these, 1260 were born to women with GDM, 152 were born to women with pre-pregnancy DM and 3783 neonates were born to healthy non-diabetic (control) women. The prevalence of GDM in the Qatari population in 2017 was 24.25%. HbA1C% before delivery was significantly higher in women with pre-pregnancy DM (mean 6.19 ± 1.15) compared to those with GDM (mean 5.28 ± 0.43) (P <0.0001). The mean birth weight in grams was 3066.01 ± 603.42 in the control group compared to 3156.73 ± 577.88 in infants born to women with GDM and 3048.78 ± 677.98 in infants born to women with pre-pregnancy DM (P <0.0001). There was no statistically significant difference regarding the mean length (P= 0.080), head circumference (P= 0.514), and rate of major congenital malformations (P= 0.211). Macrosomia (Birth weight > 4000 gm) was observed in 2.7% of the control group compared to 4.8% in infants born to women with GDM, and 4.6% in infants born to women with pre-pregnancy DM (P= 0.001). Multivariate logistic regression analysis demonstrated that higher maternal age (adjusted OR 2.21, 95% CI 1.93, 2.52, P<0.0001), obesity before pregnancy (adjusted OR 1.71, 95% CI 1.30, 2.23, P<0.0001), type of delivery C-section (adjusted OR 1.25, 95% CI 1.09, 1.44, P=0.002), and body weight to gestational age LGA (adjusted OR 2.30, 95% CI 1.64, 2.34, P<0.0001) were significantly associated with increased risk of GDM.

**Conclusion:**

Despite the multi-disciplinary antenatal diabetic care management, there is still an increased birth weight and an increased prevalence of macrosomia among the infants of diabetic mothers. More efforts should be addressed to improve the known modifiable factors such as women's adherence to the diabetic control program. Furthermore, pre-pregnancy BMI was found to be significantly associated with gestational DM, and this is a factor that can be addressed during pre-conceptional counseling.

## Background

Gestational Diabetes Mellitus (GDM) occurs in 2-9% of pregnant women worldwide and is defined as “any degree of glucose intolerance with onset or first recognition during pregnancy” [1]. During pregnancy, the placenta secretes certain diabetogenic hormones including growth hormone, corticotropin-releasing hormone, human placental lactogen, prolactin, and progesterone. Moreover, pregnancy is also associated with insulin resistance. If this insulin resistance is paired with insufficient pancreatic function, the risk of developing GDM increases [1, 2].

GDM is a major cause of pregnancy-related maternal morbidities [3]. Infants of women with diabetes mellitus (DM) have an increased risk for both large for gestational age (LGA) and preterm birth (PTB) compared with infants born to women without DM [2, 3]. Moreover, they have an increased risk of neonatal complications such as cardiovascular (CVS) and central nervous system (CNS) defects, hyperbilirubinemia, low iron stores, perinatal asphyxia, respiratory distress syndrome (RDS), hypoglycemia, hypocalcemia, polycythemia, transient hypertrophic cardiomyopathy [4–7], and macrosomia with its subsequent complications [8–12].

Due to the increasing prevalence of DM in Qatar, we wanted to revisit its impact on both maternal and neonatal populations by conducting this retrospective research study. Hence, we aimed to study the effects of both pre-pregnancy and gestational DM on growth parameters of neonates in the Qatari newborns and compare them with those of non-diabetic women (healthy control) in the same population.

## Patients and methods

The setting for this one-year population-based cohort study was the Neonatal Intensive Care Unit (NICU) of Women’s Wellness and Research Center (WWRC) in Hamad Medical Corporation (HMC), after getting the ethical/Institutional Review Board (IRB) approval from the Medical Research Center under the number MRC-01-18-041. WWRC is a large tertiary center in Doha, Qatar, with a delivery rate of over 18,000 per year. This study was conducted following institutional policies and Good Research Practice (GRP). All methods were performed following the relevant guidelines and regulations [13–15].

Data were collected between January 1, 2017, and December 31, 2017. It comprised women’s age, pre-pregnancy body mass index (BMI), gestational age at birth, placental weight, neonatal growth parameters (weight, length, and head circumference), as well as the presence or absence of major congenital malformations (CNS, CVS or gastrointestinal anomalies).

The study data were collected by the research team members from electronic patient records and clinical documentation. All collected data were kept in an excel sheet on a password-secured computer in the principal investigator's office and the principal investigator had full controlled access to the study data as per institution and ethical policies. All data were collected using anonymized format and no patient identifications were disclosed.

The target population was Qatari women with DM, either gestational or pre-pregnancy and compared them with those born to healthy non-diabetic Qatari women. We looked at the last HbA1C% (Glycated Hemoglobin) before delivery in women with GDM and those who had pre-pregnancy DM to get an idea about glycemic control in the preceding 3 months. The data of the pre-pregnancy weight were collected from the electronic patient records. For neonatal growth parameters, we used the 2013 revised Fenton growth charts standards for comparison among the groups. As per those charts, we defined SGA as < 10th percentile for weight and LGA as > 90th percentile for weight [16].

The screening, diagnosis, and management of GDM in our hospital is usually conducted by the Diabetic Team. The Diabetic Team comprises of 5 endocrinologists, 10 obstetricians, 1 ophthalmologist, 2 diabetic educators, 2 dietitians, and 6 diabetic nurses. Medical institutions in the State of Qatar screen all pregnant ladies for diabetes at the first antenatal care visit and pregnant ladies are classified accordingly. That screening is based on the 2013-WHO Criteria. It states that gestational diabetes mellitus should be diagnosed at any time in pregnancy if one or more of the following criteria are met: fasting plasma glucose 5.1-6.9 mmol/l (92 -125 mg/dl), 1-hour plasma glucose ≥ 10.0 mmol/l (180 mg/dl) following a 75g oral glucose load, or 2-hour plasma glucose 8.5-11.0 mmol/l (153 -199 mg/dl) following a 75g oral glucose load [17]. Pre-pregnancy DM was defined by either type I or type II DM before the index pregnancy. Healthy controls are women with neither Pre-pregnancy DM nor GDM. All women with positive screening tests are referred to our diabetic team for ongoing management and monitoring of gestational diabetes [18].

### Statistical analysis

Quantitative and categorical data were presented as mean ± standard deviation (SD) and frequencies (percentages). For variables that were normally distributed, differences in their mean values between two independent groups (IDM and non-IDM; major and no major congenital malformation, etc.) were compared using unpaired Student's t-test or Mann Whitney U tests as appropriate. Quantitative data between three independent groups were analyzed using one-way analysis of variance (ANOVA) or the Kruskal Wallis test as appropriate. In case of significant difference observed, the pairwise difference was compared using Bonferroni post-hoc test. Associations between two or more qualitative variables were assessed using the Chi-square (χ2) test or Fisher Exact test as appropriate. Pearson’s correlation coefficient was used to assess the strength of the linear relationship between maternal HbA1C%measured before delivery and fetal and maternal characteristics. Univariate and multivariate logistic regression analysis was applied to determine and assess the mothers’ potential risk factors and neonatal outcomes associated with the development of GDM adjusted for potential predictors and confounders such as mother’s age, BMI, type of delivery, gestational age, gender, placental weight, birth weight, macrosomia, bodyweight to gestational age, major congenital anomalies. For multivariate logistic regression models, predictor variables were considered if statistical P<0.10 level in univariate analysis or if determined a priori to be clinically important. The results of logistic regression analyses were presented as odds ratios (OR) with corresponding 95% confidence intervals (CI). Thereafter, we computed a prediction model to evaluate the discriminative ability of potentially significant variables with statistical P <0.10 on the occurrence of GDM. Pictorial presentations of the key results were made using appropriate statistical graphs. All P values presented were two-tailed, and P values <0.05 were considered statistically significant. All statistical analyses were performed using statistical packages SPSS version 27.0 (Armonk, NY: IBM Corp) and Epi-info (Centers for Disease Control and Prevention, Atlanta, GA) software.

## Results

In 2017, there were 17020 live births from 16765 deliveries, including 255 multiple pregnancies. Out of 17020 live births, 5195 babies were to Qatari women; all of these were singletons. Of these 5195 babies, 1260 were born to women with GDM, 152 were born to women with pre-pregnancy DM and 3783 neonates were born to non-diabetic (healthy control) women. Our data shows that the prevalence rate of GDM was 24.25% (95% CI 23.1, 25.4) in Qatari women.

The mean birth weight (grams) was 3066.01 ± 603.42 in the healthy control group compared to 3156.73 ± 577.88 in infants born to women with GDM and 3048.78 ± 677.98 in infants born to women with pre-pregnancy DM (Overall P <0.0001). There was no statistically significant difference among the 3 groups regarding the mean length (P= 0.080), and head circumference (P= 0.514). Macrosomia (Birth weight >4000 gm) was observed in 2.7% of the control group compared to 4.8% of infants born to women with GDM, and 4.6% of infants born to women with pre-pregnancy DM (P= 0.001). The rate of LGA infants was 13.8% in infants born to women with pre-pregnancy DM, compared to 5.7% in infants born to women with GDM, and 2% in infants born to healthy non-diabetic women (P <0.0001). On the other hand, the rate of SGA babies was 16.4% in infants born to healthy non-diabetic women compared to 10.1% in infants born to women with GDM and 10.5% in infants born to women with pre-pregnancy DM (Table 1).

**Table 1:** Maternal and neonatal characteristics of diabetic mothers and control cases

Variables	GDM Cases (n=1260)	Pre-pregnancy DM (n=152)	Control Cases (n=3783)	P-Value
**Mother’s Age (years)**	31.59 ± 5.87	34.86 ± 6.04	28.82 ± 5.67	<0.0001
**Body Mass Index**	33.80 ± 8.31	36.15 ± 6.56	31.67 ± 5.67	<0.0001
**Maternal HbA1C (%) before delivery**	5.28 ± 0.43	6.19 ± 1.15		<0.0001
**Type of Delivery**				<0.0001
Vaginal Delivery	751 (59.6%)	41 (27%)	2415 (63.8%)	
Cesarean Section	447 (35.5%)	105 (69.1%)	1116 (29.5%)	
Instrumental Delivery	62 (4.9%)	6 (3.9%)	252 (6.7%)	
**Gestational Age (weeks)**	38.12 ± 2.04	36.71 ± 2.34	38.34 ± 2.57	<0.0001
**Gender**				0.068
Male	663 (52.6%)	65 (42.8%)	1969 (52%)	
Female	597 (47.4%)	87 (57.2%)	1814 (48%)	
**Placental Weight (gm)**	664.70 ± 120.02	643.95 ± 126.42	650.19 ± 167.31	0.013
**Birth Weight (gm)**	3156.73 ± 577.88	3048.78 ± 677.98	3066.01 ± 603.42	<0.0001
**Macrosomia (Weight >4000 gm at term)**	60 (4.8%)	7 (4.6%)	103 (2.7%)	0.001
**Length (cm)**	49.85 ± 3.40	49.24 ± 3.30	49.73 ± 3.29	0.080
**Head Circumference (cm)**	34.12 ± 1.81	33.93 ± 2.13	34.05 ± 2.56	0.514
**Body weight to Gestational Age**				
SGA	127 (10.1%)	16 (10.5%)	617 (16.4%)	<0.0001
AGA	1061 (84.2%)	115 (75.7%)	3080 (81.7%)	0.0104
LGA	72 (5.7%)	21 (13.8%)	74 (2%)	<0.0001
**Major Congenital Anomalies**				0.211
No	1252 (99.4%)	149 (98%)	3753 (99.2%)	
Yes	8 (0.6%)	3 (2%)	30 (0.8%)	

The mean gestational age (weeks) of neonates born to healthy non-diabetic women was significantly higher than those born to women with pre-pregnancy DM and GDM mothers (P <0.0001). The pre-pregnancy Body Mass Index (BMI) was significantly higher (P <0.0001) in women with pre-pregnancy DM (36.15 ± 6.56) and women with GDM (33.80 ± 8.31) compared to healthy non-diabetic women (31.67 ± 5.67). The placental weight (grams) was found to be significantly higher in women who had GDM (mean 664.70 ± 120.02) than the other 2 groups (P=0.013) (Table 1).

The rate of caesarian section, in healthy non-diabetic women, was significantly lower than in women with pre-pregnancy DM and women with GDM (P <0.0001). On the other hand, instrumental delivery was significantly higher in the control group compared to the other groups (P <0.0001). (Table 1).

A total of 41 infants were born with major congenital malformations in 2017, of Qatari women. Interestingly, major congenital malformations were seen in only 0.6% of the infants of women with GDM, compared to 0.8% in healthy control, and 2% of infants born to women with pre-pregnancy DM (P= 0.211) (Table 1). Those anomalies included 26 cases with Congenital Heart Disease (CHD), 9 cases of GIT malformations, 3 cases of CNS malformations, and 3 cases of multiple congenital anomalies. The presence of congenital malformation was not significantly associated with any of the neonatal or maternal characteristics (Table 2).

**Table 2:** Maternal and neonatal characteristics of infants born with and without major congenital malformation

Variables	Presence of major congenital malformation (n=41)	No major congenital malformation (n=5154)	P-Value
**Mother’s Age (years)**	31.46 ± 6.70	29.66 ± 5.90	0.51
**DM Status**			0.211
GDM	8 (0.6%)	1252 (99.4%)	
Pre-pregnancy DM	3 (2%)	149 (98%)	
Controls	30 (0.8%)	3753 (99.2%)	
**Body Mass Index**	33.70 ± 6.37	32.31 ± 6.53	0.180
**Maternal HbA1C (%) before delivery**	5.66 ± 0.49	5.40 ± 0.65	0.202
**Type of Delivery**			0.446
VD	24 (0.7%)	3183 (99.3%)	
CS	16 (1%)	1652 (99 %)	
ID	1 (0.3%)	319 (99.7%)	
**Gestational Age (weeks)**	38.78 ± 1.351	38.23 ± 2.470	0.157
**Gender**			0.687
Male	20 (0.7%)	2677 (99.3%)	
Female	21 (0.8%)	2477 (0.8%)	
**Placental Weight (gm)**	645.98 ± 111.72	653.58 ± 156.46	0.756
**Birth Weight (gm)**	3142.56 ± 527.60	3087.08 ± 601.40	0.556
**Length (cm)**	49.61 ± 3.33	49.75 ± 3.32	0.789
**Head Circumference (cm)**	34.05 ± 1.82	34.06 ± 2.39	0.974
**Body weight to Gestational Age**			0.960
SGA	6 (0.8%)	754 (99.2%)	
AGA	4 (0.8%)	4222 (99.2%)	
LGA	1 (0.6%)	166 (99.4%)	

The mean maternal glycosylated hemoglobin (HbA1C%%) before delivery was 6.19 ± 1.15 in women with pre-pregnancy DM, compared to 5.28 ± 0.43 in women with GDM (P <0.0001). Pearson correlation analysis showed maternal glycosylated hemoglobin (HbA1C%%) before delivery had a significantly high positive correlation with the mother’s age (Pearson r= 0.70, P=0.017). However, both BMI (Pearson r =0.12, P<0.001) birth weight (Pearson r =0.11, P<0.001) had a significant but weakly positive correlation with maternal HbA1C%. In contrast, the correlation between gestational age and maternal HbA1C% showed an inverse and weak correlation (Pearson r = -0.13, P<0.0001).

**Fig. 1:**
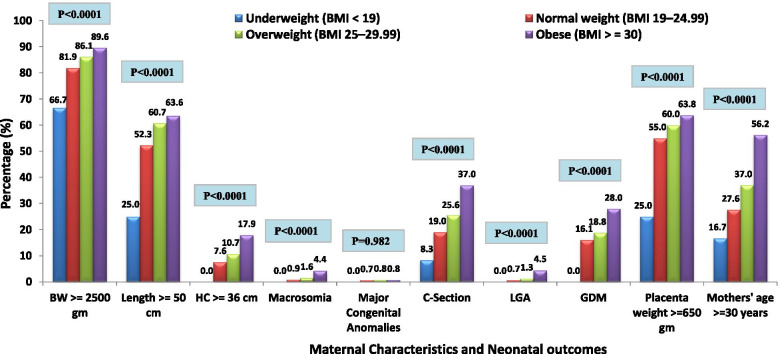
Maternal Characteristics and Pregnancy and Neonatal outcomes across various BMI categories

**Fig. 2:**
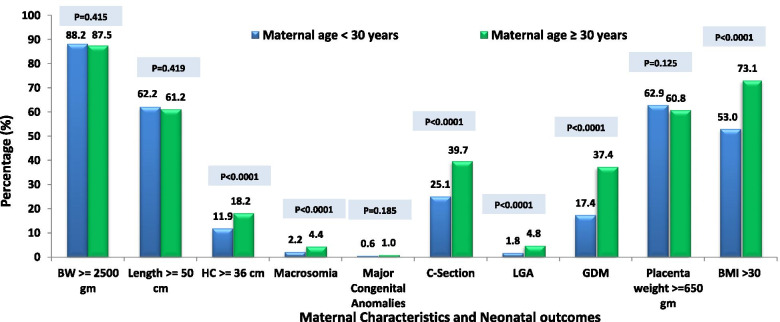
Maternal Characteristics and Pregnancy and Neonatal outcomes across maternal age categories

There were significant associations between maternal characteristics and pregnancy and neonatal outcomes across various BMI categories. A significant increasing trend was observed when compared to both maternal and neonatal outcomes in obese and overweight with those in normal and underweight groups (P<0.0001). However, pre-pregnancy overweight and obesity didn’t affect major congenital anomalies (P=0.982) (Fig. 1). As depicted in Fig. 2, there were significant associations observed between maternal characteristics and pregnancy and neonatal outcomes with maternal age categories. Maternal age ≥ 30 years was significantly associated with a higher rate of HC ≥ 36 cm, macrosomia, C-section, LGA, GDM, and BMI>30 (P<0.0001). Whereas birth weight, length, major congenital anomalies, and placental weight showed insignificant differences between maternal age categories (P>0.05) shown in Fig. 2.

The results of univariate and multivariate logistic regression analysis testing for each predictor and their possible association with GDM and neonatal outcomes are presented in Tables 3 and 4. Compared to mothers of normal weight (BMI between 19 and 24.99), noted before pregnancy or at the first visit, mothers with a BMI between 25 to <30, had an increased risk of developing GDM (unadjusted OR 1.21, 95% CI 0.91, 1.61; P=0.192). The risk of developing GDM in women who were obese before pregnancy was two folds higher than that in the group of normal weight (unadjusted OR 2.12, 95% CI 1.62, 2.76, P<0.0001). The higher maternal age (≥30 years) was significantly associated with the increased risk of developing GDM compared to the age group <30 years (unadjusted OR 2.38, 95% CI 2.09, 2.71, P<0.0001). When examined for the association between GDM (Table 3) and birth weight, higher birth weight (≥2500 gm) (unadjusted OR 1.39, 95% CI 1.13, 1.72; P=0.002) was significantly higher in the GDM group compared to control group. The presence of GDM significantly increases the risk of C-sections (unadjusted OR 1.29, 95% CI 1.12, 1.48; P<0.0001) however, the difference in instrumental delivery in the two groups were statistically insignificant (P=0.113). Women with GDM delivered babies with a higher proportion (approximately three-fold) of ‘large for gestational age’ infants than women with AGA (unadjusted OR 2.82, 95% CI 2.03, 3.94; P<0.0001). The newborns of women with GDM were at a twofold increased risk of being macrosomic (unadjusted OR 1.79, 95% CI 1.29, 2.47, P<0.0001). We did not observe that GDM significantly influenced gestational age at birth (≥37 weeks) and major congenital anomalies (P>0.05) as shown in Table 3.

**Table 3:** Factors associated with GDM: Univariate Logistic regression analysis

Variables	GDM n/N (%)	Unadjusted Odds ratio (OR)	95% CI for OR	P-value
**Mother’s Age (years)**				
**<30**	463/2658 (17.4%)	1.0 (reference)		
**≥30**	797/2385 (33.4%)	2.38	2.09, 2.71	<0.0001
**Body Mass Index (BMI)**				
**Normal weight (19-24.99)**	71/437 (16.2%)	1.0 (reference)		
**Overweight (25-29.99)**	278/1463 (19%)	1.21	0.91, 1.61	0.192
**Obese (≥30)**	910/3125 (29.1%)	2.12	1.62, 2.76	<0.0001
**Type of Delivery**				
**Vaginal Delivery**	751/3166 (23.7%)	1.0 (reference)		
**Cesarean Section**	447/1563 (28.6%)	1.29	1.12, 1.48	<0.0001
**Instrumental Delivery**	62/314 (19.7%)	0.79	0.59, 1.06	0.113
**Gestational Age (weeks)**				
**<37**	155/583 (26.6%)	1.0 (reference)		
**≥37**	1105/4460 (24.8%)	0.91	0.75, 1.11	0.342
**Gender**				
**Male**	663/2632 (25.2%)	1.0(reference)		
**Female**	597/2411 (24.8%)	0.98	0.86, 1.11	0.726
**Placental Weight (gm)**				
**<650**	448/1904 (23.5%)	1.0 (reference)		
**≥650**	806/3122 (25.8%)	1.13	0.99, 1.29	0.069
**Birth Weight (gm)**				
**<2500**	120/603 (19.9%)	1.0 (reference)		
**≥2500**	1140/4438 (25.7%)	1.39	1.13, 1.72	0.002
**Macrosomia**				
**No**	1200/4878 (24.6%)	1.0 (reference)		
**Yes**	60/163 (36.8%)	1.79	1.29, 2.47	<0.0001
**Bodyweight to Gestational Age**				
**AGA**	1061/4141 (25.6%)	1.0(reference)		
**SGA**	127/744 (17.1%)	0.60	0.49, 0.73	<0.0001
**LGA**	72/146 (49.3%)	2.82	2.03, 3.94	<0.0001
**Major Congenital Anomalies**				
**No**	1252/5005 (25%) 8/38 (21.1%)	1.0 (reference)		
**Yes**		0.80	0.37, 1.75	0.575

*CI* Confidence interval, *OR* Odds ratio; Outcome variable: non-GDM was considered as the reference group

*LGA* Large for gestational age, *AGA* appropriate for gestational age, *SGA* small for gestational age

‘n’ is the total number of GDM cases whereas ‘N’ is the total number of participants included against each specific variable/parameter

**Table 4:** Factors associated with GDM: Multivariate Logistic regression analysis

Variables	GDM n/N (%)	Adjusted Odds ratio (OR)	95% CI for OR	P-value
**Mother’s Age (years)**				
**<30**	463/2658 (17.4%)	1.0 (reference)		
**≥30**	797/2385 (33.4%)	2.21	1.93, 2.52	<0.0001
**Body Mass Index (BMI)**				
**Normal weight (19-24.99)**	71/437 (16.2%)	1.0 (reference)		
**Overweight (25-29.99)**	278/1463 (19%)	1.11	0.83, 1.48	0.493
**Obese (≥30)**	910/3125 (29.1%)	1.71	1.30, 2.23	<0.0001
**Type of Delivery**				
**Vaginal Delivery**	751/3166 (23.7%)	1.0 (reference)		
**Cesarean Section**	447/1563 (28.6%)	1.25	1.09, 1.44	0.002
**Instrumental Delivery**	62/314 (19.7%)	0.82	0.61, 1.20	0.176
**Bodyweight to Gestational Age**				
**AGA**	1061/4141 (25.6%)	1.0(reference)		
**SGA**	127/744 (17.1%)	0.70	0.57, 0.87	0.001
**LGA**	72/146 (49.3%)	2.30	1.64, 3.24	<0.0001

*CI* Confidence interval, *OR* Odds ratio, Outcome variable: non-GDM was considered as the reference group

*LGA* Large for gestational age, *AGA* appropriate for gestational age, *SGA* small for gestational age

‘n’ is the total number of GDM cases whereas ‘N’ is the total number of participants included against each specific variable/parameter

The multivariable logistic regression analysis indicated that higher maternal age (adjusted OR 2.21, 95% CI 1.93, 2.52, P<0.0001), obesity before pregnancy (adjusted OR 1.71, 95% CI 1.30, 2.23, P<0.0001), type of delivery C-section (adjusted OR 1.25, 95% CI 1.09, 1.44, P=0.002), and body weight to gestational age LGA (adjusted OR 2.30, 95% CI 1.64, 2.34, P<0.0001) remained significantly associated with increased risk of GDM adjusting all other potential confounder and predictors (Table 4). Therefore, we computed a prediction model to evaluate the discriminative ability of potentially significant variables with statistical P <0.10 on the occurrence of GDM. Multivariate logistic regression (stepwise variable selection approach) indicated that the final model demonstrated a modest fit (area under the curve (AUC) = 0.633, 95% CI 0.62, 0.65) and included the following variables maternal age and BMI before pregnancy as shown in Table 4.

## Discussion

The prevalence rate of GDM in the Qatari population in our study sample was 24.25%, which is higher than the rates observed by Bener A. et al (16.3%) [19] in 2011 but close to the rates observed by Bashir M. et al (23.5%) [20] in 2016. Studies from neighboring countries such as Oman and Bahrain reported a lower prevalence (10%) of GDM [21, 22].. Our prevalence is also higher than the rates observed in Kuwait (12.6%) [23] and in the United Arab Emirates (UAE) (13.3%) [21]. On the other hand, in Saudi Arabia, the prevalence of GDM ranged between 24% and almost 40% [24–27]. The relatively high prevalence of GDM in Qatar might be related to overweight or obesity, excessive weight gain during pregnancy, excessive central body fat deposition, positive family history of DM, and the relatively sedentary lifestyle and high socio-economic standard in Qatar compared to other populations [28].

Despite the overall statistically significant difference in the mean birth weight values among the three groups, the mean birth weight difference between infants born to women with GDM and healthy non-diabetic mothers was only 90 gm. The data from this study shows no statistically significant difference in length and head circumference in babies born to women with GDM in comparison with those who were born to healthy non-diabetic women. Other studies also did not find any difference in birth measures between the GDM-exposed and unexposed neonates [29, 30]. On the other hand, Baptiste-Roberts K. et al [31] concluded that compared to their non-diabetic counterparts, mothers with GDM gave birth to offspring that had higher weights at birth even after adjustment for other variables (β = 50 gm; 95% CI: 0.01, 0.09). Moreover, Sletner L et al [32] found that offspring of GDM mothers were smaller in mid-pregnancy but subsequently grew faster until birth, compared with offspring of non-GDM mothers.

Another interesting result in our study was the rate of macrosomia which was only 4.8% among infants born to Qatari women with GDM and 4.6% among infants born to Qatari women with pre-pregnancy DM, as compared to a previous study on the same population ( 9.3%) [20]. A literature review by Kc K. et al. [33] concluded that about 15–45% of babies born to diabetic mothers can have macrosomia, which is a 3-fold higher rate when compared to normoglycemic controls.

Macrosomia is expected in 20% of infants born to women whose postprandial glucose values average 120 mg/dL or less [34]. However, this rate can be higher (35%) when postprandial levels in women range as high as 160 mg/dL [35]. However, other studies showed different results. For instance, Vally F. et al [31] reported that macrosomia is not increased in women with diet-controlled GDM in comparison with healthy controls, and others reported low percentages of macrosomia (8-14%) among IDM mothers [36, 37].

As expected, the mean gestational age of neonates born to women with pre-pregnancy DM was significantly lower than those of GDM and healthy non-diabetic women. However, there was no difference between the incidence of preterm labor between GDM women and healthy controls. In contrast to our findings, Billionnet C. et al [38] found that the risks of preterm birth (OR 1.3 [95% CI 1.3, 1.4]) were increased in women with GDM compared with the non-diabetic population. In addition to Köck K. et al [39] also reported a significant difference in the incidence of spontaneous preterm birth (P = 0.047) between IDMs and healthy controls. The reasons for an increased risk of spontaneous preterm delivery are not clear [40, 41], but could be explained by the fast-intrauterine growth due to overexposure to the energy source [37].

Researchers believe that the hyper-insulinemic state in IDMs accompanied by the upregulation of gene expression, inflammatory mediators, and leptin in placental tissues could be the cause of excessive growth and an increase in placental weight [42]. In our study, the placental weight of neonates born to women with GDM was significantly higher than those of healthy non-diabetic women. Daskalakis et al. [43] compared the placentas of healthy pregnant women against GDM patients and had similar findings to ours. They found degenerative changes such as fibrinoid necrosis, cholangitis, and the presence of nucleated fetal erythrocytes, in addition to villous immaturity as an indicator of chronic fetal hypoxia. Macroscopically, they found the fetal/placental weight ratio was significantly decreased. On the other hand, Akarsu S. et al [44] concluded that no significant difference was found between the groups in terms of fetal/placental weight ratio.

Major congenital malformations were found in 0.6% of infants born to GDM women, compared to 0.8% in healthy controls and 2% in women with pre-pregnancy DM. The literature has reported that the overall reported risk for major malformations is approximately 5 to 6 percent with a higher prevalence rate of 10 to 12 percent when mothers require insulin therapy [45–47]. Out of the 41 cases of major congenital anomalies in our study, 29 involved the cardiovascular and central nervous systems. Becerra JE et al [45] found that two-thirds of the anomalies in infants of mothers with diabetes involve either the cardiovascular system (8.5 per 100 live births) or central nervous system (CNS) (5.3 per 100 live births) [45]. Those malformations were not significantly associated with any maternal or fetal parameters. Moreover, Prakash GT et al reported a 2.3% (3/132) rate of major congenital anomalies in infants born to GDM women [48].

HbA1C% is an important indicator and prognostic factor of long-term blood sugar control with the ability to reflect the cumulative blood sugar history of the preceding three months. HbA1C% level below 5.7% is considered normal [49]. In our study, the last HbA1C% results before delivery were obtained in diabetic mothers. Its mean value was 5.28 ± 0.43 in women with GDM and 6.19 ± 1.15 in women with pre-pregnancy DM (P <0.0001). A significant positive correlation with HbA1C% results was observed with maternal age, BMI, and birth weight while a significant negative correlation with HbA1C% results was observed only with gestational age. Our correlation findings are similar to those of Sweeting AN et al who stated that baseline HbA1C%>5.9% (41 mmol/mol) identifies an increased risk of large-for-gestational-age, macrosomia, cesarean section, and hypertensive disorders in standard GDM [47, 48]. Nevertheless, a 2013 systematic review and meta-analysis of randomized trials for the US Preventive Services Task Force found that reductions in pre-eclampsia, macrosomia, and shoulder dystocia were associated with appropriate management of GDM [50].

Risk for large for gestational age and congenital anomalies are higher in women with elevated HbA1c% levels during pregnancy [51]. However, the HbA1C% mostly associated with congenital anomalies is the one taken in the periconceptional period that is not known in our study. Achieving near normal levels of HbA1C% before delivery reflects the proper antenatal care and the socioeconomic standard of the population which subsequently led to proper control of GDM despite the high BMI before delivery. While this paper does not focus on identifying risk factors for diabetes in pregnancy, the high risk of overweight and obesity in Qatar is indeed a likely major contributing factor to the high prevalence of diabetes in pregnancy. Despite recent advances in its diagnosis, follow-up, and management, GDM continues to be a common complication of pregnancy and a cause of great concern because of the relatively high rates of adverse maternal, fetal and neonatal outcomes. The study outcomes emphasize the importance of collaboration between feto-maternal medicine and neonatology in weighing the fetal and maternal risks of prolonged pregnancy compared to the potential benefits of further fetal maturation among most gestational ages.

The main limitation of the study is being a retrospective cohort with no long-term follow-up outcomes. However, the number of the population tested increases the significance of its results. Getting deeper insights about the periconceptional levels of HbA1C% in the diabetic population might further lower the rate of major congenital anomalies as well as other neonatal and maternal morbidities. Understanding the pathophysiology of the disorder may allow the development of strategies and routine screening measures to prevent morbidities in those babies. Hence, studying the molecular pathogenesis of neonatal morbidities related to GDM is recommended using prospective studies with a larger sample size and long-term outcomes measurements.

## Conclusion

Despite the multi-disciplinary antenatal diabetic care management, there is still an increased birth weight and an increased prevalence of macrosomia among the infants of diabetic mothers. More efforts should be addressed to improve the known modifiable factors such as women's adherence to the diabetic control program. Furthermore, pre-pregnancy BMI was found to be significantly associated with gestational DM, and this is a factor that can be addressed during pre-conceptional counseling.

## Data Availability

The datasets used and/or analyzed during the current study are available from the corresponding author on reasonable request.

## Abbreviations

GDM: Gestational Diabetes Mellitus
DM: Diabetes Mellitus
IDM: Infant of Diabetic Mother
PTB: Preterm Birth
HbA1C: Glycosylated Hemoglobin
LGA: Large for Gestational Age
BMI: Body Mass Index
SGA: Small for Gestational Age
AGA: Appropriate for Gestational Age
CVS: Cardiovascular System
CNS: Central Nervous System
WHO: World Health Organization
VD: Vaginal Delivery
CS: Cesarean Section
ID: Instrumental Delivery
GA: Gestational Age
BW: Birth Weight
AUC: Area Under the Curve
OR: Odds Ratio
CI: Confidence Interval
WWRC: Women’s Wellness and Research Center
IRB: Institutional Review Board
HMC: Hamad Medical Corporation
NICU: Neonatal Intensive Care Unit
MRC: Medical Research Center
