# Correction to: Maternal and neonatal outcomes in mothers with diabetes mellitus in Qatari population

**DOI:** 10.1186/s12884-021-04244-z

**Published:** 2021-11-16

**Authors:** Mohammad A. A. Bayoumi, Razan M. Masri, Nada Y. S. Matani, Mohamed A. Hendaus, Manal M. Masri, Prem Chandra, Lisa J. Langtree, Sunitha D’Souza, Noimot O. Olayiwola, Saad Shahbal, Einas E. Elmalik, Mohamed S. Bakry, Ashraf I. Gad, Ravi Agarwal

**Affiliations:** 1 Neonatal Intensive Care Unit (NICU), Women’s Wellness and Research Center (WWRC), Hamad Medical Corporation (HMC), P.O. Box 3050, Doha, Qatar; 2 Department of Medical Education, Hamad Medical Corporation (HMC), Doha, Qatar; 3 Pediatric Department, Sidra Medicine, Doha, Qatar; 4 Al-Ahli Hospital, Doha, Qatar; 5 Medical Research Center, Hamad Medical Corporation (HMC), Doha, Qatar; 6 Medical Records Department, Women’s Wellness and Research Center (WWRC), Hamad Medical Corporation (HMC), Doha, Qatar; 7 Corporate Communications Department, Hamad Medical Corporation (HMC), Doha, Qatar; 8 Obstetrics and Gynecology Department, Faculty of Medicine, Fayoum University, Fayoum, Egypt

**Correction to: BMC Pregnancy Childbirth 21, 651 (2021)**

**https://doi.org/10.1186/s12884-021-04124-6**

Following publication of the original article [1], the authors reported an error in Tables 2 and 3. Below are the details.

In table 2: The percentage should be changed from 0.8 to 99.2% in the female row under the gender. This is really significant.

In table 3: The number and percentage 8/38 (21.1%) should be opposite to yes not in the same line with no in the last row of major congenital anomalies. This is also significant.

The original article [1] has been updated.
